# Numerical Optimization of a Nanophotonic Cavity by Machine Learning for Near-Unity Photon Indistinguishability at Room Temperature

**DOI:** 10.1021/acsphotonics.1c01651

**Published:** 2022-05-11

**Authors:** J. Guimbao, L. Sanchis, L. Weituschat, J. Manuel Llorens, M. Song, J. Cardenas, P. Aitor Postigo

**Affiliations:** † Instituto de Micro y Nanotecnología, IMN-CNM, CSIC (CEI UAM+CSIC), Tres Cantos, Madrid E-28760, Spain; ‡ The Institute of Optics, University of Rochester, Rochester, New York 14627, United States

**Keywords:** single-photon, neural network, genetic algorithm, nanophotonics, nanocavity

## Abstract

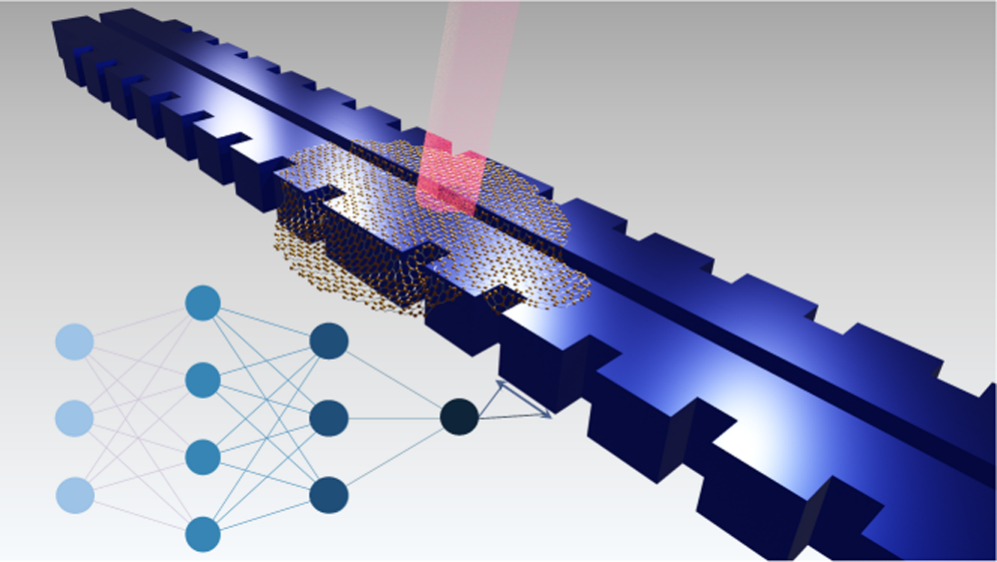

Room-temperature (RT), on-chip deterministic generation of indistinguishable photons coupled to photonic integrated circuits is key for quantum photonic applications. Nevertheless, high indistinguishability (*I*) at RT is difficult to obtain due to the intrinsic dephasing of most deterministic single-photon sources (SPS). Here, we present a numerical demonstration of the design and optimization of a hybrid slot-Bragg nanophotonic cavity that achieves a theoretical near-unity *I* and a high coupling efficiency (β) at RT for a variety of single-photon emitters. Our numerical simulations predict modal volumes in the order of 10^–3^(λ/2n)^3^, allowing for strong coupling of quantum photonic emitters that can be heterogeneously integrated. We show that high *I* and β should be possible by fine-tuning the quality factor (*Q*) depending on the intrinsic properties of the single-photon emitter. Furthermore, we perform a machine learning optimization based on the combination of a deep neural network and a genetic algorithm (GA) to further decrease the modal volume by almost 3 times while relaxing the tight dimensions of the slot width required for strong coupling. The optimized device has a slot width of 20 nm. The design requires fabrication resolution in the limit of the current state-of-the-art technology. Also, the condition for high *I* and β requires a positioning accuracy of the quantum emitter at the nanometer level. Although the proposal is not a scalable technology, it can be suitable for experimental demonstration of single-photon operation.

## Introduction

Indistinguishable single photons are the leading candidates for quantum communication and quantum information processing technologies. They play a central role in a range of proposed schemes, including quantum simulation,^1^ quantum walks,^2^ boson sampling,^3^ quantum teleportation,^4^ and quantum networks.^5^ However, the complex mesoscopic environment of solid-state sources entails fundamental barriers that restrict the operation to cryogenic temperature (*T*).^6^ Trying to overcome the thermal restrictions of quantum devices remains a challenge for the development of on-chip, on-demand single-photon sources (SPS). A feasible approach for achieving efficient indistinguishable photon emission from a solid-state emitter consists of maximizing the emitter–field coupling (*g*) through the effective confinement of light in an ultrasmall cavity-mode volume (*V*_eff_) and reaching the strong coupling regime.^7^ In this regime, the transfer rate between the emitter and the cavity field exceeds the dephasing rate of the emitter, and the emitted photons are able to leave the cavity before being affected by decoherence.^7^ Plasmonic cavities with subnanometer gaps between dimers like Au spheres,^8^ Ag nanowires,^9^ and surface plasmon-polariton systems^10^ or metallic bowties with CdSe/ZnS quantum dots^11^ produce the highest *g* value up to 200 meV^11^ and the lowest quality factors (*Q* ∼ 10).^9^ There are different proposals to improve *Q* and β in these systems, some of them involving dielectric-core/metal-shell schemes for *Q*(10,12) or hybrid FP-nanoantenna cavities for β.^13,14^ However, using plasmonic cavities faces two obstacles:^15^ (i) the placement of the emitter in the point with the strongest cavity field can be challenging and (ii) ohmic and quenching losses can be very high. The use of dielectric cavities can avoid the latter limitation, and strong coupling can happen using strategies to decrease the modal volume, like slotted photonic crystals. Discrete slotted nanobeams^16,17^ lead to volumes in the order of 10^–3^(λ/2n)^3^ while keeping high *Q*. However, because introducing a finite slot causes a large perturbation to the optical mode, β values remain low. Continuous-slot designs improve β and *Q*,^18^ and more recently, slot–antislot concatenations in 1D-PC^19,20^ have shown record *Q*/*V*_eff_ ratios with PC cavities. Also, designs based on cascaded cavities schemes have shown promising results with dielectric structures.^21^ According to those works, a slotted dielectric cavity can provide a sufficient small modal volume for strong coupling, thus a high *I*, avoiding at the same time the losses inherent to plasmonic cavities. However, for highly dissipative emitters, the dependence of *I* with *g* at RT is highly nontrivial.^7^ With high *g*, there is a high population transfer rate between the emitter and the cavity field, so the emitted photons must leave the cavity before getting dephased by the emitter. This can be accomplished by setting the right *Q*. As we will show, this trade-off between different rates (i.e., dephasing rate, *g*, and *Q*) translates into a complex dependence of *I* with the cavity figures of merit.

In this work, we show that achieving a high *I* at RT requires tuning of *Q* together with a small modal volume. That does not translate to a high *Q* but a specific *Q* threshold depending on the emitter’s intrinsic properties and the modal volume. From our calculations, none of the previously mentioned dielectric cavities can provide a high *I* for strong dissipative emitters despite achieving small modal volumes. Furthermore, the implementation of machine learning algorithms for the geometrical optimization of the cavity modal volume and *Q* has shown promising results in recent works.^22−25^ Here, we present a numerical demonstration of a design strategy for high indistinguishable SPS at RT strongly coupled to a hybrid slot-Bragg waveguide cavity. We vary the geometrical parameters of the waveguide cavity (i.e., the waveguide width, slot width, number of periods), and we obtain a theoretical estimation of the cavity performance for *I*, β, and the Purcell enhancement. We explore different types of promising SPS (InGaAs^26^ and GaAs^27^ quantum dots, single molecules,^28^ localized excitons in transition metal dichalcogenides transition-metal dichalcogenide (TMDC) monolayers,^29^ and diamond color centers^30^), and we obtain theoretical near-unity *I* and high β simultaneously by parameter optimization. Finally, we develop a hybrid deep neural network-genetic algorithm (GA) scheme that further reduces the modal volume for achieving near-unity *I* with a slot width of 20 nm. The optimized device presents strong challenges for current fabrication and quantum emitter (QE) positioning techniques. In this regard, we have developed a comparison of the design requirements with the state-of-the-art demonstrations.

## Methods

We can compute the value of *I* for a QE with radiative decay rate γ and pure dephasing rate γ* coupled to a photonic cavity (with decay rate κ and electromagnetic coupling constant *g*) from the Lindblad equation and applying the quantum nonregression theorem. For each (*g*, κ, γ, γ*), we have:^7^
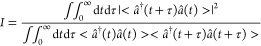
1where *â*^†^ and *â* are the creation and annihilation operators of the cavity mode, respectively. Details of the calculation can be found in the Supporting Information. The values of *g* and κ are linked to *Q* and *V*_eff_ by κ ∼ 1/*Q* and *g* ∼ 1/√*V*_eff_.

**Figure 1:**
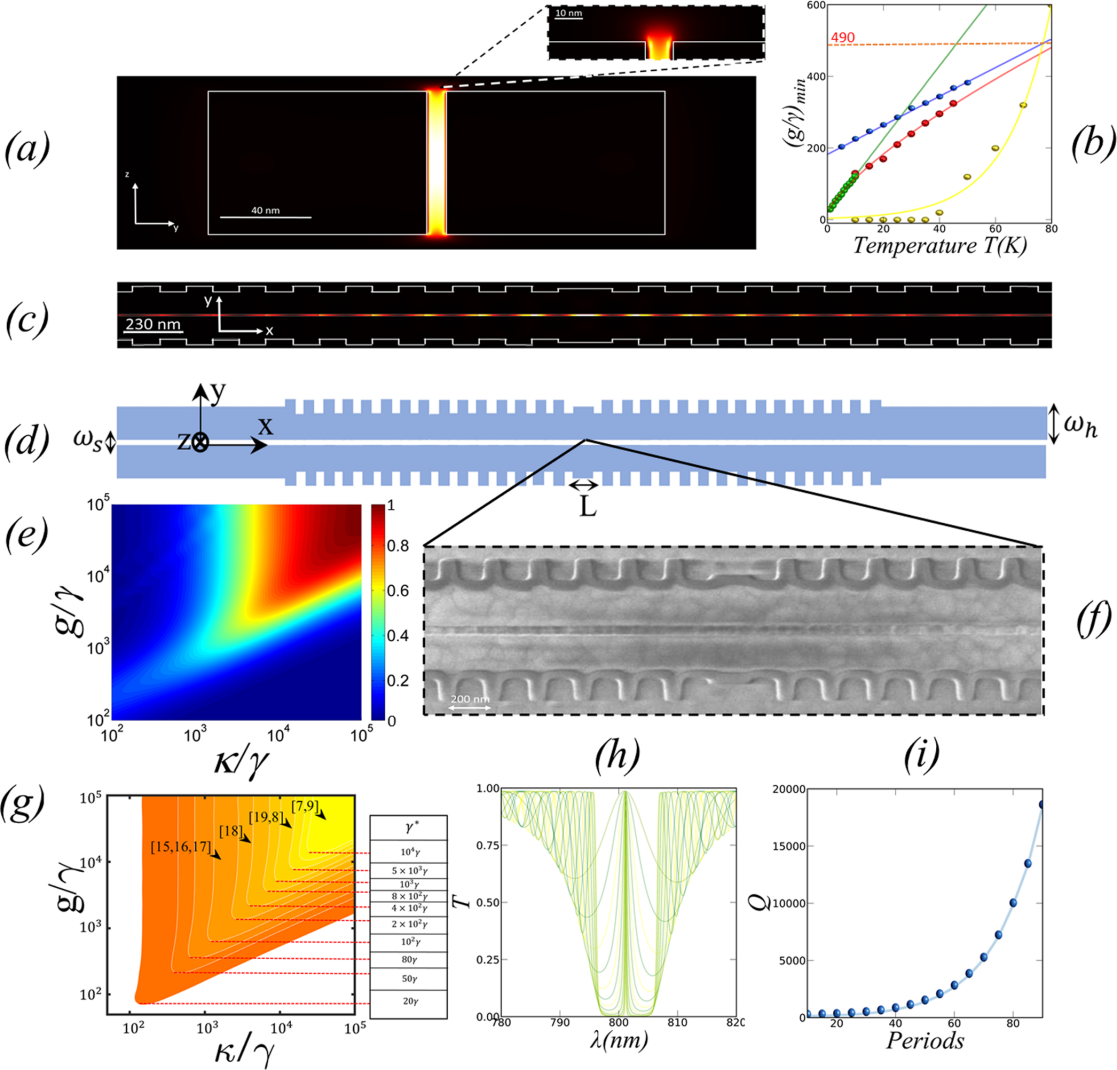
(a) |*E*|^2^ field profile in the *y*–*z* plane. (b) Variation of the ratio (*g*/γ)_min_ with *T* for *I* > 0.9 and different SPS: GaAs (red), S.molecules (green), two-dimensional (2D) materials (blue), InAs (yellow). (c) |*E*|^2^ field profile of the cavity mode in the *x*–*y* plane. (d) Layout of the proposed structure, where ω_h_ is the width of each waveguide, ω_s_ is the slot width, *L* is the cavity length, and Λ is the grating period. (e) Color map of *I* as a function of *g*/γ and κ/γ for photons emitted by a high dissipative QE with γ* = 10^4^γ. (f) SEM image of the center of the cavity. (g) Contour map of regions with *I* > 0.9 for different dephasing values (γ* = 20γ, 50γ, 80γ, 10^2^γ, 2 × 10^2^γ, 4 × 10^2^γ, 8 × 10^2^γ, 10^3^γ, 5 × 10^3^γ, and 10^4^γ). (h) Transmission spectrum of the structure for a different number of periods, the full width at half maximum (FWHM) of the resonance scales exponentially with #*p*. (i) *Q* versus number of periods.

Figure 1e shows the value of *I* for photons emitted by a high dissipative QE with γ* = 10^4^ γ as a function of *g* and κ normalized to γ in the coherent strong-coupling regime (i.e., *g* > γ* + γ). In this regime, the rate of photon transfer from the emitter to the cavity is *R* = 4*g*^2^/κ,^7^ which exceeds the pure dephasing rate (*R* > γ*) for certain values of κ. For a high *I*, the photon must escape out of the cavity before the emitter dephases it. In other words, κ > γ*, which means that a small *Q* is needed. Specifically, for a QE with γ* = 10^4^γ, one needs a value of κ/γ above 2 × 10^4^ for *I* > 0.9. The region of high *I* in Figure 1e has a shape and area that depend on *T* through γ*. For a QE at RT, γ*∼ 10^4^ γ^7^ and the minimum value of *g*/γ to achieve *I* > 0.9 is (*g*/γ)_min_ ∼ 10^4^. As γ*/γ decreases, the area of high *I* grows and (*g*/γ)_min_ decreases.

Figure 1g shows the contour maps of the region with a high *I* (*I* > 0.9) as γ* changes. For moderate dissipative emitters (γ* ∼ 10^2^ γ), the minimum *g*/γ necessary for *I* > 0.9 is (*g*/γ)_min_ = 1 0^3^. As γ* increases (*g*/γ)_min_ grows monotonously, reaching 10^4^ for γ* ∼ 10^4^ γ. Similarly, the minimum (κ/γ)_min_ increases from 10^3^ for γ* ∼ 10^2^, to 2 × 10^4^ for γ* ∼ 10^4^ γ. We can use this color map to plot the cavities mentioned before, according to its performance for *I*. Plasmonic cavities^8−10^ can achieve *I* > 0.9 even for high dissipative emitters with γ* ∼ 10^4^ γ. On the other hand, slotted dielectric cavities^16−18^ can achieve *I* > 0.9 for emitters with γ* between ∼10^2^ to ∼2 × 10^2^ γ and slot–antislot concatenations in 1D-PC^19^ for emitters with γ* ∼ 2 × 10^2^ γ to γ* ∼ 4 × 10^2^ γ. The cavity shown in ref (20) is the only one, in the group of dielectric structures, that can reach *I* > 0.9 when γ* > 2 × 10^3^ γ. According to our calculations, those dielectric cavities can potentially achieve the region with *I* > 0.9 for high dissipative emitters (i.e., QE at RT) just by increasing its cavity decay rate κ (i.e., deteriorating its quality factor *Q*). Figure 1b shows the dependence of the value (*g*/γ)_min_ with *T* for *I* > 0.9, calculated for quantum dots of GaAs^31^ and InAs,^32^ organic molecules,^33,34^ and defects in 2D materials.^35^ The evolution of (*g*/γ)_min_ with *T* shows a proportional increase with a different trend that depends on γ*. We can obtain the (*g*/γ)_min_ needed for *I* > 0.9 for a QE at a specific *T* from Figure 1g. It is interesting to observe that for the technologically relevant *T* of liquid nitrogen (77 K), the same value (*g*/γ)_min_ = 490 works for InAs and GaAs QDs and 2D materials.

Therefore, our goal is to keep the κ/*g* ratio inside the region with a high *I* by increasing *g* and adjusting *Q*. Moreover, we look for an on-chip cavity that can be CMOS-compatible with photonic integrated circuits (PICs) used in silicon photonics. Slotted one-dimensional dielectric photonic crystal cavities^16−20^ have been shown to fulfill most of our requirements in terms of compatibility and small modal volume. Nevertheless, to efficiently control *Q*, we choose a hybrid slot-Bragg cavity, where *Q* changes by the number of periods of the Bragg reflector section. Figure 1d shows a layout of our hybrid slot-Bragg photonic cavity aiming to achieve a near-unity *I* and a high β simultaneously; ω_h_ is the width of each waveguide, ω_s_ is the slot width, and #*p* is the number of periods. While this structure has been explored for sensing applications,^36−38^ it has never been proposed for SPS operation, as far as we know, nor its performance is calculated in terms of the figures of merit (*I*, β). It consists of a phase-shifted corrugated Bragg grating situated at the sides of a Si_3_N_4_ (*n*_1_ = 2) deposited on top of a SiO_2_ substrate (*n*_2_ = 1.4). The cavity length *L* corresponds to the central section between the two periodic regions and matches the wavelength of the zero-order Fabry–Perot mode for the target wavelength λ. The Si_3_N_4_ thickness (*t*) is set for optimum field enhancement at the slot for the target λ. Each of the periodic regions behaves like a mirror with an effective reflectivity that depends on the number of periods (#*p*), creating a Fabry–Perot structure. The grating period Λ matches the central frequency of the photonic bandgap at the target λ. To get information about the physical behavior of the device, we will set first λ = 801 nm to perform a general evaluation of the performance. After that, for each type of emitter, the geometrical parameters of the device (i.e., *t*, *L*, and Λ) are set to match the specific emission wavelength λ: (λ, *t*, *L*, Λ) = (915, 900, 263, 263 nm) for InGaAs,^26^ (916, 900, 263, 263 nm) for GaAs,^27^ (728, 710, 210, 210 nm) for TMDC,^28^ (785, 770, 225, 225 nm) for S.molecules,^29^ and (685, 680, 195, 195 nm) for diamond color centers.^30^Figure 1a shows how the slotted cross section of the cavity enhances the field of the zero-order TE mode in the gap showing an evanescent tail in the top of the waveguide. This field distribution provides advantages related to the coupling of the source when it is heterogeneously integrated on top. The cavity provides strong coupling if the slot width is sufficiently small, and it also provides advantages in extraction efficiency (β) since (i) cavity and output waveguide share the same cross section, so the modes are perfectly matched; (ii) the integration of the QE (for example colloidal QDs) can be done by direct deposition on top of the cavity, which avoids interferences by total internal reflection and enhances β; and (iii) the slot mode has the field maxima at the edges of the slot, which matches well with the region of maximum probability of having SPS in 2D materials deposited on top of waveguides.^39^ Finally, the cavity modal volumes are in the order of 10^–3^(λ/2*n*)^3^ along with the whole slot, increasing the probability of having one or several QE strongly coupled to the cavity mode. As a proof of concept, we have fabricated a specific design valid for diamond color center requirements. We selected (ω_s_, #*p*) = (38 nm, 50) and added vertical grating couplers to the structure to collect the input and output light beams. Figure 1f shows an SEM image of the cavity fabricated by e-beam lithography (EBL) and reactive-ion etching on a layer of 130 nm thick Si_3_N_4_ deposited on top of a SiO_2_ layer (1 μm thick) by plasma-enhanced chemical vapor deposition (PECVD). The obtained slot width is ω_s_ = 54 nm, and the grating period is 204 nm, with less than 5% of the error to the initial design for the grating period and 30% for ω_s_. According to our simulations, the wider slot translates into a modal volume increase, *V*_eff_ ∼ 6 × 10^–2^(λ/2n)^3^, which slightly reduces the indistinguishability to *I* = 0.81. This issue can be solved by further optimization by machine learning, as we will show later. We can obtain the transmission spectrum *T*(λ) shown in Figure 1h and the field profile (Figure 1c) of the cavity mode for a set (ω_s_, ω_h_, #*p*) using a fully vectorial, bidirectional, frequency-domain model for solving Maxwell’s equation (3D-FD).^40^ We obtain *Q* from *T*(λ) by  and the cavity decay rate from κ = ω/2*Q*. Details of the model appear in the Supporting Information. There is a different effective index for each set (ω_s_, ω_h_, #*p*), so the values of Λ and *L* are changed to keep the cavity resonance at 801 nm. The volume of the cavity-mode *V*_eff_ is^41^
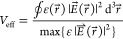
2

**Figure 2:**
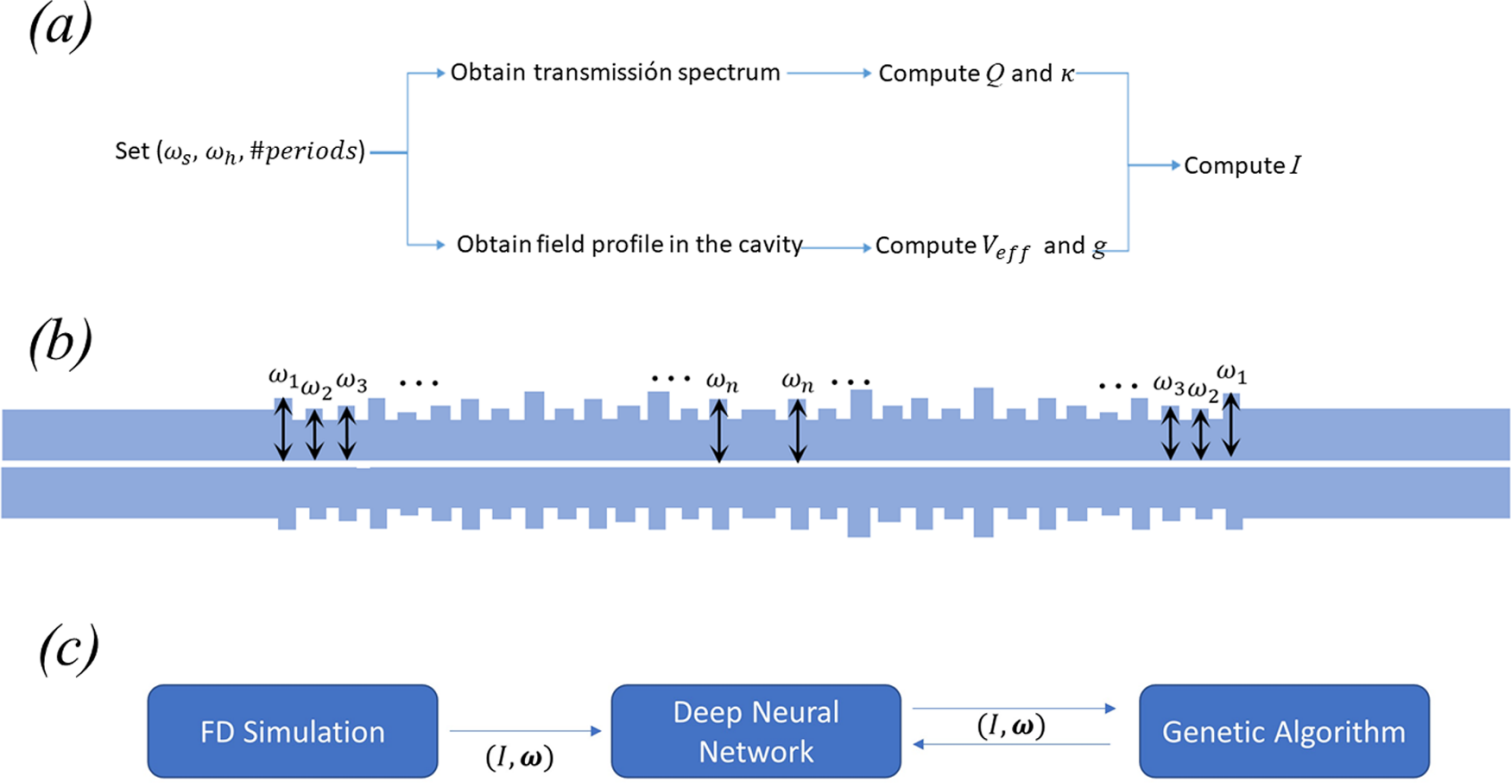
(a) Outline of the computation algorithm for the calculation of *I.* (b) Parametrization of the Bragg corrugations for machine learning optimization. Each ω_i_ represents the width of the corresponding Bragg corrugation. (c) Routine for the hybrid NN-GA optimization of the Bragg corrugations.

The value of *g*, when the QE is placed at the maximum cavity field and perfectly matches the polarization, is^42^
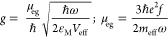
3where μ_eg_ is the electric dipole moment of the excitonic transition, ω is the frequency of the transition, *e* is the electron charge, ε_M_ is the dielectric constant in the source region, ℏ is the reduced Planck constant, *m*_eff_ is the exciton effective mass, and *f* is the oscillator strength. Once we have *g* and κ, we obtain *I* according to the procedure outlined in Figure 2a. For the computation of the Purcell enhancement (Γ_p_) and the coupling efficiency β, we perform three-dimensional-finite difference time domain (3D-FDTD) simulations^40^ by placing a dipole point source emitting at 801 nm with position *x*_0_, *y*_0_ at the center of the slot and *z*_0_ 4 nm above the top of the waveguides. We obtain Γ_p_ by integrating the power *P* emitted by the source and normalizing it to the power inside a homogeneous environment *P*_0._(43) Finally, we calculate β by measuring the fraction of light coupled to guided modes at the output waveguide. Details of the simulations appear in the Supporting Information.

Our design strategy can be further enhanced using machine learning techniques, especially to keep critical fabrication parameters, like the slot width ω_s_, experimentally accessible and far from too narrow and unrealistic values. Recently, the optimization of nanophotonic structures by deep learning techniques has been reported.^22−25^ The two main advantages are: (i) further improved performance beyond the time-consuming method of sweeping the (ω_s_, ω_h_, #*p*) parameters and (ii) we can introduce a vast number of new parameters for the optimization, such as the width of each of the Bragg corrugations, as shown in Figure 2b.

For that purpose, we can use a vector **ω** = (ω_1_, ω_2,_ ω_3_,...,ω_*n*_), where each entry ω_*i*_ with *i* = 1,...,20 represents the width of each Bragg corrugation. For each configuration **ω**, we obtain *I* using the two-step method described in Figure 2c. We use a GA to create a random vector **ω** and the fitness function obtains *I* from the 3D-FD simulation (Figure 2a). Through the iteration of crossover and mutation, the GA should find the optimal configuration for maximizing *I* after a certain number of generations. Details of the code appear in the Supporting Information. However, this procedure faces a critical issue. Typically, in a GA optimization, one needs to generate about 10^5^ pairs (**ω**, *I*) and the generation of each pair (**ω**, *I*) involves a 3D-FD simulation that may take several minutes, making the whole optimization process unfeasible in terms of time and computational resources. To solve this issue, we take a different approach: (i) we generate 5000 pairs (**ω**, *I*) through 3D-FD simulations; (ii) with these data, we train a deep neural network (NN) which learns to estimate the outcome of *I* for any possible **ω**. Now we can use the NN to calculate *I* for the fitness function of the GA optimization. In this way, the calculation of the fitness function for each **ω** takes just a few seconds; (iii) We perform the GA optimization by calculating the fitness function for each individual of the population through the NN. With this scheme, we reduce by 2 orders of magnitude the number of actual numerical simulations for the dataset from 10^5^ to 10^3^ with the aid of the NN.

## Results and Discussion

**Figure 3:**
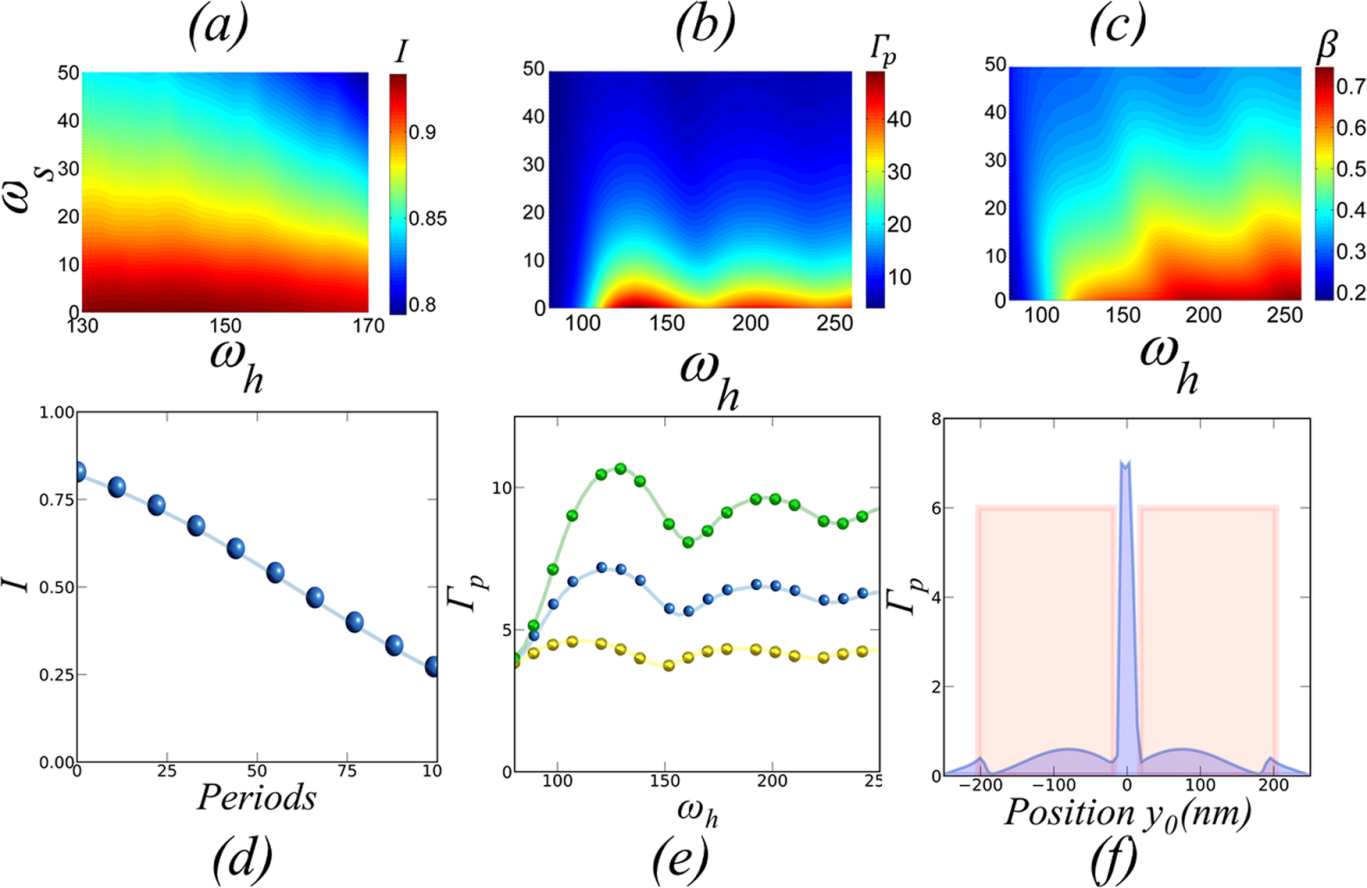
(a) Cavity-induced *I* when γ/γ* = 10^4^ versus waveguide width (ω_h_) and slot width (ω_s_) for #*p* = 10. (b) Purcell enhancement (Γ_p_) versus waveguide width (ω_h_) and slot width (ω_s_). (c) Coupling efficiency (β) versus waveguide widt_*h*_ (ω_h_) and slot width (ω_s_) for #*p* = 10. (d) *I* versus number of grating periods (#*p*) for (ω_s_, ω_h_) = (5 nm, 140 nm). (e) Γ_p_ versus ω_h_ for three ω_s_ (green, ω_s_ = 15 nm; blue, ω_s_ = 20 nm*;* yellow, ω_s_ = 25 nm). (f) Γ versus source position *y*_0_ along the *y*-axis.

We first assess the performance of the cavity by sweeping the main geometrical parameters and setting a target λ = 801 nm; *t*, *L*, and Λ are set to (*t*, *L*, Λ) = (800, 230, 230 nm), respectively. Figure 3 shows how *I* changes with (ω_s_, ω_h_) and #*p* when γ* = 10^4^ γ (a typical ratio for many QE at RT as we have seen before). Figure 3a shows *I* versus ω_h_ and ω_s_ for #*p* = 10 with ω_s_ varying between 10–50 nm and ω_h_ between 150–220 nm (required for single-mode operation). With #*p* fixed, *Q* remains constant (*Q* = 50), while the field profile of the cavity mode varies for each (ω_h_, ω_s_). Therefore, the variation of *I* follows the variation of *g* with ω_h_ and ω_s_. As ω_s_ increase, the cavity mode spreads out from the slot and gets confined at each waveguide core separately. That results in an exponential decay of the field intensity in the slot region,^44^ increasing *V*_eff_ exponentially with ω_s_. Since *g* ∼ 1/√*V*_eff_, *g* decreases, driving the system to the weak coupling regime (i.e., going downward in Figure 1a) and inducing an exponential decay of *I*. For a small enough ω_s_ (<20 nm), the system remains in the strong-coupling regime and *I* becomes independent of *g*.^7^ Therefore, we can observe that for ω_s_ < 20 nm, *I* shows a weak variation with ω_h_. When ω_s_ > 20 nm, the cavity starts to perform away from the strong coupling regime and *I* shows an evident change with ω_h_, which we will further analyze later. A slot width ω_s_ <10 nm produces a maximum value of *I* = 0.96, decaying with ω_s_ at a rate of 5 × 10^–3^ nm^–1^. Figure 3d shows the dependence of *I* with #*p*, with #*p* in the range of 10–100 and fixed ω_h_ = 140 nm and ω_s_ = 15 nm so we keep the strong coupling regime. As #*p* increases, the effective reflectivity also increases and the *Q* factor grows exponentially (see Figure 1i). Consequently, κ decreases exponentially with #*p*. Therefore, the time that the photon stays in the cavity increases exponentially with #*p*, and when κ < γ*, the photon is dephased by the emitter (i.e., going in the left direction in Figure 1a). The result is that *I* decreases with #*p* giving *I* = 0.4 for #*p* = 100. Figure 3b shows Γ_p_ versus (ω_s_, ω_h_) when #*p* = 10, ω_s_ in the range 10–100 nm, and ω_h_ between 110 and 600 nm. Since Γ_p_ ∼ 1/*V*_eff_, Γ_p_ changes with ω_s_ in a similar way to *I*. As the slot mode spreads over the waveguide cores, the field’s intensity at the source’s position decreases and Γ_p_ shows an exponential decay. The change with ω_h_ displays a more complex structure, shown more clearly in Figure 3e. For ω_s_ = 15 nm and ω_h_ = 80, Γ_p_ increases monotonically as the zero-order cosine/even^45^ slot mode gets more efficiently confined in the waveguide. Γ_p_ is maximum (Γ_p_ = 11) when ω_h_ = 125 nm, and the strongest light confinement in the waveguide happens. For a higher ω_h_, the mode spreads over the structure producing a decay of the overlapping with the source that scales with 1/ω_h_. The decay interrupts abruptly when the zero-order sin-type/odd mode cutoff is reached at ω_h_ = 155 nm. From there, the same pattern reproduces until the activation of the subsequent mode, and so on. The same behavior happens for ω_s_. However, as ω_s_ increase the dependence of Γ_p_ with ω_h_ shifts to lower values of ω_h_. This is because the ω_h_ cutoff value of the zero-order sine mode/odd decreases monotonically with ω_s._^45^ Therefore, the activation of the second mode shifts to lower values of ω_h_ as ω_s_ increases.

Figure 3c shows β versus ω_s_ and ω_h_ for the same values of #*p*, ω_s_ and ω_h_ used in Figure 3b. While Γ_p_ is a measure of the field enhancement due to the overlapping of all available modes, β accounts just for the overlapping with guided modes. Therefore, we expect a similar dependence and, in fact, β shows an exponential decay with ω_s_ similarly to *I* and Γ_p_. The dependence with ω_h_ shows the same “mode jumps” found for Γ_p_, giving a maximum β = 75% at ω_h_ = 128 nm. In this case, the regions of high β become bigger for higher values of ω_h_, as the number of available modes increases with ω_h_.

The position of the QE inside the cavity plays a relevant role.^46^ To explore the effect of the position of the QE in Γ_p_, we have performed 3D-FDTD simulations changing the position (*y*_0_) of the QE along the cavity cross section (*y*-axis) at *z*_0_ = 4 nm above the top of the cavity. Figure 3f shows Γ_p_ versus *y*_0_ varying from −225 to +225 nm when ω_h_ = 200 nm, ω_s_ = 30 nm, and #*p* = 10. Since Γ_p_ is proportional to the field of the available modes for each spatial position, the plot reproduces the field profile of the zero-order mode of the slot waveguide. The maximum Γ_p_ happens in the region inside the slot, with maxima at the edges of the waveguides. The enhancement falls abruptly inside the waveguide, with values reduced by 1 order of magnitude. For a QE located away from the outer edges of the waveguide cores, the evanescent coupling increases the enhancement slightly. In summary, even for a strong dissipative emitter with γ* = 10^4^γ, we can achieve *I* > 0.9 by adjusting the number of periods and reducing the slot width ω_s_ below 10 nm. At the same time, a high Purcell enhancement (Γ_p_ = 45) and a good extraction efficiency (β = 0.7) can be obtained for the same ω_s_. On the other hand, we need an accurate positioning of the emitter inside the slot region.

We further explore the performance of the device and the design requirements for different types of QE with different dephasing rates. For each type of emitter, the geometrical parameters of the device (i.e., *t*, *L*, and Λ) are set to match the specific emission wavelength λ. Table 1 shows the values of the pairs (ω_s_, #*p*) needed for *I* > 0.9 for five different γ*/γ values corresponding to each emitter. The values of the oscillation strengths are extracted from InGaAs,^47^ GaAs,^48^ TMDC,^49,50^ single molecules,^28,51^ and diamond.^52^ We observe that as γ* increases (i.e., *T* increases), the cavity demands smaller ω_s_ (i.e., narrower slot). For the highest oscillator strength (∼5 in InGaAs QD and diamond color centers), (*g*/γ)_min_ is easily reached when ω_s_ < 44 nm and γ* = 10^2^γ. A TMDC QE with oscillator strength ∼ 0.1 demands ω_s_ < 38 nm on the opposite side. In an intermediate situation, the oscillation strength of the GaAs QD (∼1) gives ω_s_ < 42 nm. From this, we can find the optimal design for each emitter at a high *T*. InGaAs at 300 K has a pure dephasing of 600γ,^53^ so (ω_s_, #*p*) = (36 nm, 50) are needed for *I* > 0.9. GaAs at 300 K has 1450γ^54^ and needs the same values (ω_s_, #*p*) = (36, 50). High dissipative emitters with dephasing of ∼10^4^γ at 300 K, like TMDC^55^ and single molecules, demand narrower slot widths (ω_s_, #*p*) = (5 nm, 10). For color centers in diamond, with γ* = 10^3^γ at room T,^56^ the optimal configuration is (ω_s_, #*p*) = (38 nm, 50).

**Table 1:** Maximum (ω_s_ (nm), #*p*) for *I* > 0.9 Using InGaAs QD, GaAs QD, TMDCs, and Single Molecules as QE

	γ* = 10^2^γ	γ* = 10^3^γ	γ* = 10^4^γ
InGaAs	(43,100)	(36,50)	(15,10)
GaAs	(41,100)	(30,50)	(9,10)
TMDC	(36,120)	(25,60)	(5,12)
S.molecules	(40,120)	(28,60)	(8,12)
Diamond	(45,100)	(38,50)	(15,10)

**Figure 4:**
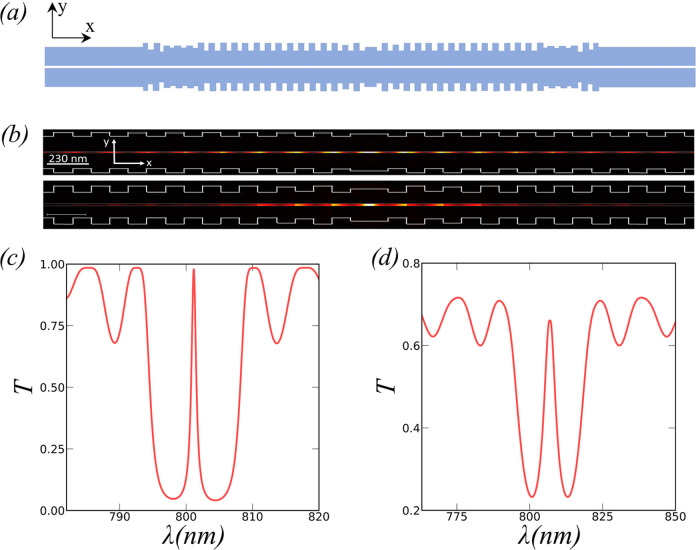
(a) Optimized structure for fixed (ω_s_, #*p*) = (20,20). Cavity-mode field profile in the *XY* plane inside the cavity region for (b) cavity mode profiles of the nonoptimized structure (top) and optimized structure (bottom). Transmission spectra for (c) structure without optimization and (d) GA-NN optimized structure.

As we have shown, for high dissipative emitters with γ* = 10^4^γ, the width of the cavity slot must be ω_s_ < 10 nm for *I* > 0.9. Similarly, ω_s_ < 10 nm is needed for β > 0.7. At the same time, the emitter’s position plays a critical role, giving very low coupling when the emitter is outside the slot region. These requirements make complex both the fabrication and the emitter integration. Achieving slot widths below 10 nm is beyond the state of the art of almost any fabrication technology, and deterministic deposition of a QD with that accuracy can be complicated. To reduce those limitations, we need to optimize the geometry of the cavity further. We have performed a hybrid GA-NN optimization of the Bragg corrugation geometry. The GA-NN optimization must deal with the trade-off between reducing the cavity modal volume (to increase *g*) and maintaining the appropriate *Q* to achieve *I* > 0.9 with γ* = 10^4^γ. With this aim, we set ω_s_ = 20 nm and the number of periods to #*p* = 20. The structure without optimization has a modal volume of about 10^–2^(λ/2n)^3^, which gives *I* = 0.82 with γ* = 10^4^γ. Figure 4a shows the GA-NN optimized geometry. Somehow surprisingly to us, the GA-NN found that it is enough to change the widths of the most external Bragg corrugations, leaving the others unperturbed. This geometry provides the best confinement of the cavity mode in the center of the structure, significantly reducing the modal volume while maintaining the correct *Q*.

Figure 4b–d shows the cavity-mode profile and the transmission spectrum for the structure with and without optimization. It is easy to appreciate how the cavity mode is significantly more confined in the central region of the optimized cavity. The modification of the widths of the external Bragg corrugations creates a tapered section that connects the cavity with the input/output slot waveguides and increases the confinement of the cavity mode. The modal volume is reduced from 7 × 10^–3^(λ/2n)^3^ to 2.5 × 10^–3^(λ/2n)^3^, a factor of 2.8. At the same time, FWHM has been increased to *Q* = 50, keeping the system in the region of high *I*. The reduction in the modal volume and the *Q* adjustment improve the indistinguishability from *I* = 0.82 to 0.91. In conclusion, we obtain that for the optimized structure, we can achieve *I* > 0.9 for γ* = 10^4^γ with a slot width of ω_s_ = 20 nm, relaxing the tight requirements for the fabrication of the slot to more realistic values. The resulting transmission spectra of the optimized device reveal that there is a 7 nm shift of the resonance wavelength. This results from the discontinuous alteration of the periodicity of the Bragg reflectors. The λ-Bragg condition for total reflection changes along the corrugations, giving rise to a small modification of the spectra. This resonance displacement could be reduced through a second optimization process involving the maximization of *I* together with the minimization of the λ-shift, which will be covered in future works.

Although simulation results show a promising device performance, potential difficulties related to fabrication have to be considered according to CMOS-compatible processes. Realization of vertical slot widths below 80 nm can be difficult with standard lithography techniques. For emitters with γ* = 10^2^γ, slots between 36 and 45 nm are needed (see Table 1). Despite that achieving these widths can be challenging, there are many experimental demonstrations reporting the fabrication of sub-100 nm slots (between 30 and 80 nm) using e-beam lithography (EBL).^57−64^ On the other hand, strong dissipative emitters with γ* = 10^4^γ require slot widths between 5 and 15 nm. Defining sub-10 nm structures with EBL is a great challenge, requiring simultaneous control of several factors like resist contrast, beam diameter, resist development mechanics, and limitations in metrology.^65^ A novel fabrication procedure with EBL was reported,^65^ which allows us to achieve slot widths down to 8 nm in Si substrates. Also, recent works^66^ experimentally demonstrate a different fabrication approach achieving slots with 10 nm width in Si waveguides. In this context, the relaxation up to 20 nm width achieved through the ML optimization is especially relevant since it reduces the fabrication requirements from the limit of the technology (5 nm) to a more accessible value (20 nm). Still, we must emphasize that achieving such ultranarrow slots presents a significant challenge, which requires top-state-of-the-art resolution technology.

Another key aspect to consider for the experimental realization is the nanoscale positioning approach for the deposition of the QE in the 20 nm slot region of the cavity. Recently, several nanoscale positioning techniques compatible with nanofabrication processes have shown promising results, achieving positioning accuracy at the nanometer level.^67^ Atomic force microscopy-based positioning approaches with 30 nm positioning accuracy have been reported with GaAs QDs strongly coupled to a nanocavity.^68^ Confocal micro-photoluminescence techniques also showed 10 nm positioning accuracy with GaAs QDs inside a photonic structure.^69^ Bichromatic photoluminescence approaches with 5 nm position accuracy were recently achieved through a novel image analysis software implementation in the positioning setup.^70^ Also, in situ lithographic techniques, where the QD position extraction and the nanostructure definition are developed in the same setup, have improved position accuracy down to 30 nm.^71^ Pick-and-place techniques, which are the most suitable approach for our specific structure, have also shown significant progress.^72^ Recently, Si vacancy centers were transferred to AIN waveguides achieving 98% coupling efficiency,^73^ the placement mean error was about 38 nm. According to this, for a pick-and-place deposition, assuming a normal distribution, we would have a standard deviation of 38 nm with a target of 20 nm, which leads to 34% probability of successful deposition. Therefore, the positioning accuracy required for our structure lies close to the limit of the technology depending on the positioning approach. An experimental realization of a QE coupling requires fabricating many devices and looking for good candidates one by one. This approach allows the experimental demonstration of certain quantum effects for quantum information applications, but is still far from a scalable technology.

## Conclusions

We explored a hybrid slot-Bragg nanophotonic cavity for the generation of indistinguishable photons at RT from various quantum emitters through a combination of numerical methods. We obtain the values of the theoretical indistinguishability, efficiency, and Purcell enhancement for each configuration (i.e., waveguide width, slot width, number of periods). We obtained theoretical near-unity indistinguishability and high efficiency simultaneously by parameter sweep optimization. To relax the fabrication requirements (slot width) for near-unity indistinguishability, we have developed a machine learning algorithm that provides the optimal geometry of the cavity. According to our simulations, the optimized structure shows high indistinguishability (*I* > 0.9) with slot widths of about 20 nm. The geometrical features of the optimized design present significant challenges from the perspective of fabrication process. Although the device may be far from a real scalable technology, it can be suitable for experimental demonstration of single-photon operation. Also, the developed ML approach may provide insights for the optimization of different photonic structures for quantum information applications.

## Supporting Information Available

Computation of the indistinguishability; numerical simulation; machine learning algorithm; and fabrication process (PDF)

## Notes

Details underlying the results presented in this paper are available in the Supplement 1 file.
