# Supplementary material for: Numerical Optimization of a Nanophotonic Cavity by Machine Learning for Near-Unity Photon Indistinguishability at Room Temperature
Source: ACS Photonics. 2022 May 11;9(6):1926–35. doi: 10.1021/acsphotonics.1c01651 (PMC9205277; doi:10.1021/acsphotonics.1c01651)
Supplement: Supplementary file 1 — ph1c01651_si_001.pdf [file ph1c01651_si_001.pdf]

Numerical optimization of a nanophotonic cavity by machine learning for near-unity photon indistinguishability at room temperature: supplemental document

J. GUIMBAO[*1], L. SANCHIS[1], L. WEITUSCHAT[1], J. MANUEL LLORENS[1], M. SONG[2], J. CARDENAS[2], P. AITOR POSTIGO[1,2]

[1]*Instituto de Micro y Nanotecnología, IMN-CNM, CSIC (CEI UAM+CSIC), Tres Cantos, Madrid, E-28760 Spain*
[2] *The Institute of Optics, University of Rochester, Rochester, New York 14627, USA*
**j.guimbao@csic.es*

Number of pages: 5

Number of figures: 2

A. Computation of the indistinguishability

Our system consists of a quantum emitter $\{|g\rangle,|e\rangle\}$ coupled to a single-mode-cavity mode $\{|0\rangle, |1\rangle\}$. The parameters for the emitter are the radiative decay rate γ and the pure dephasing rate γ^* $\sim 10^4\ \gamma$ (a typical value for a solid-state QE at room temperature[1]). The parameters for the cavity are the cavity decay rate κ, and the electromagnetic coupling constant g. The detuning between the cavity and the emitter is neglected. We consider instantaneous excitation of the emitter, so the total energy during the whole process is restricted to one quanta. We truncate the system removing the states with no coupling with the dynamics, obtaining the two-dimensional Hilbert space: $\{|e\rangle,0\rangle,|g\rangle,1\rangle\}$. After rotating-wave-approximation, the Hamiltonian reads:

$$H = \hbar\omega_{QE}\hat{e}^\dagger\hat{e} + \hbar\omega_c\hat{a}^\dagger\hat{a} + g(\hat{e}\hat{a}^\dagger + \hat{e}^\dagger\hat{a}) \qquad \text{(A1)}$$

In the Born-Markov approximation, the evolution of the density matrix follows the Lindblad equation:

$$\frac{\partial\rho}{\partial t} = -i[H,\rho] + \sum_n \left(D_n\rho D_n^\dagger - \frac{1}{2}(D_n^\dagger D_n\rho + \rho D_n^\dagger D_n)\right) \qquad \text{(A2)}$$

Where the D_n denotes the collapse operators: $\sqrt{\kappa}\hat{a}$, $\sqrt{\gamma}\hat{e}$ and $\sqrt{\gamma^*}\hat{e}^\dagger\hat{e}$. The degree of indistinguishability of photons is defined using equation (A1) in the main text and is computed applying quantum non-regression theorem.

B. Numerical simulations

The simulation method to get transmission $T(\lambda)$ and cavity field profile is based on a fully vectorial, bi-directional, frequency domain model for solving Maxwell's equations (EME solver from Lumerical MODE solutions package)[2]. The steps followed by this solver are the following:

(i) The structure is divided along the propagation direction (x-axis in Figure S1b) in a set of individual domains called cells.

(ii) At each cell, it computes the corresponding waveguide modes by solving Helmholtz equation in the YZ cross section. After it performs a modal decomposition of the input electromagnetic fields into the eigenmodes basis.

(iii) Scattering matrices for each cell interface are then formulated by matching the tangential E and H fields at the cell boundaries.

(iv) The solution to each section can be propagated bi-directionally to calculate the S matrix of the entire device and the internal fields are also reconstructed in 3D.

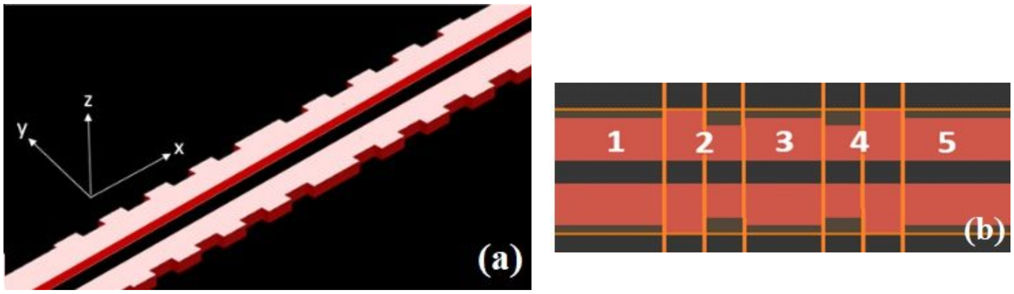

Fig S1. Lay-out of the simulation set-up for the calculation of transmission spectrum and cavity mode-profile

For the computation of the Purcell enhancement Γ_p and the coupling efficiency β we performed 3D Finite Difference Time Domain (FDTD) simulations using the layout of Figure 3a in the

main text. A dipole point source with 800 nm emission wavelength was placed at the center of the cavity inside the gap of the slot waveguide. The simulation general mesh size was set to 10 nm, whereas the specific size in the area surrounding the dipole source was set to 1 nm. The total size of the simulation was 10x10x10 μm^3, and all boundary conditions were set to Perfect Matched Layer. The Purcell enhancement can be obtained integrating the power emitted by the source inside the cavity P and normalizing respect to the power in a homogeneous environment P_0[3]. For $\frac{P}{P_0}$ calculation the Fourier transformed Pointing vector was integrated over a 10^3 nm squared box surrounding the source and then normalized with respect the total emission in a homogeneous environment. The calculation of β was performed by measuring the fraction of light coupled to guided modes at the output slot waveguide. For that we integrated the pointing vector Fourier transform over the cross section of the output slot waveguide and normalized with respect the total emission injected in the simulation.

C. Genetic Algorithm and Deep Neural Network

Our NN consists in a sequential layer model implemented in Keras module with the corresponding settings: Number of layers = 6; Neurons per layer = 1000; input-dimension = 20; output dimension = 1; loss = mean square error; Epochs = 200; learning rate = 0.0001; Batch size = 500; Number of samples = 4650. After the training with 4650 samples both loss and validation-loss converged to 10^{-4}, giving enough accuracy for the estimation of I and the optimization model. The Genetic Algorithm uses decimal representation for the genes, one point crossover and uniform mutation. The total initial population was set to 3000, the number of parents mattings =1000, number of weights = 1000, and we needed 300 generations to find the optimal geometry. The estimation for the optimal indistinguishability was 0.96, very close to the value of 0.91obtained introducing the optimal geometry in the 3D-FD simulation.

D. Exploration of the thickness

To explore the behavior of the indistinguishability versus (ω_s, ω_h), we have to ensure that the slot waveguide has the right thickness (d) to provide low loss single-mode operation at λ = 800 nm. In particular, the window of the study is (ω_s=[0-50 nm], ω_h=[130-170 nm]). Within this range, the thickness must lie below the cutoff thickness of the second-order mode while maintaining low loss propagation of the first order mode. We performed numerical simulations using the Lumerical MODE eigensolver[2] to find the available modes and their properties depending on the waveguide geometry.

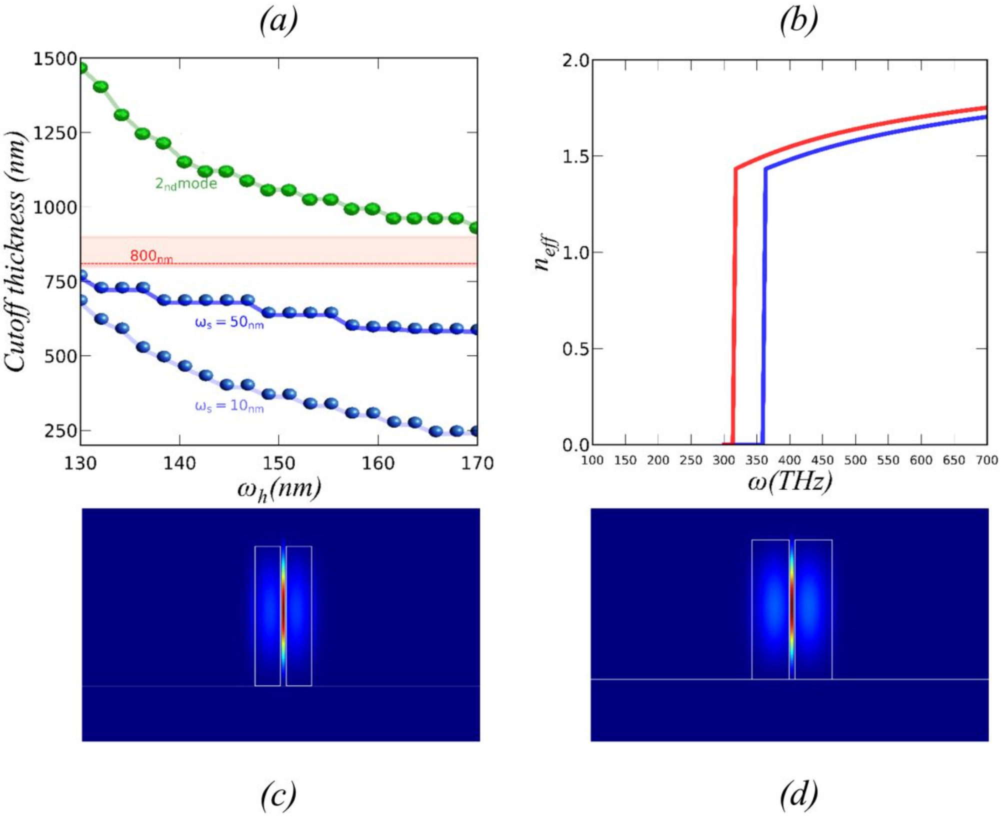

Fig S2. (a) Cutoff thickness of the first cos-type mode for two different ω_s (blue balls) and second order sin-type mode (green balls) versus ω_h. (b) Effective index versus the mode propagation frequency, ω_h = 130 nm (red line) and ω_h = 170 nm (blue line) for the selected thickness. Mode profile of the first order cos-type mode for (c) ω_h = 130 nm, (d) ω_h = 170 nm.

Figure S2a shows the cutoff thickness of the first order cos-type mode (blue balls) and second order sin-type mode (green balls) versus ω_h and for two different ω_s. As ω_h increases the cutoff thickness of all the modes decreases from a maximum of d = 765nm (first mode, second mode) at ω_h = 130 nm down to d = 615nm at ω_h = 170 nm. On the other hand, the cutoff thickness grows monotonically with ω_s [4] from d = 698nm at (ω_s, ω_h) = (10, 130) nm to d =765 nm at (ω_s, ω_h) = (50, 130) nm. This means that the right thickness must lie inside the red area in Fig 2.a. We found d = 800 nm the best option to ensure single mode operation while having low loss propagation (0.08 dB/cm and 0.01 dB/cm for ω_h = 130 nm and ω_h = 170 nm, respectively). Also, this thickness value provides the best field confinement inside the slot. Figure S2b shows the effective index n_{eff} variation with the mode propagation frequency ω for the selected thickness (ω_h = 130 nm (red line) and ω_h = 170 nm (blue line)). The cutoff frequencies are well below our frequency of operation (374 THz for λ = 800 nm) and the n_{eff} is about 1.5. Figures S2c,d show the mode profiles for d = 800 nm and, ω_h = 130 nm and ω_h = 170 nm, respectively. It can be seen that in both cases the mode is well confined inside the slot with almost negligible leaking to the substrate. The thickness value of d = 800 nm is a commonly used geometry especially in quantum photonic applications. Three representative examples found in the literature are: Epping [5] with slot Si_3N_4 waveguides of d = 900 nm, Kippenberg's [6] with d = 1350 nm and Lipson[7] with d = 910 nm.

E. Fabrication process

As is shown in Figure S2a, the cutoff thickness of the first order mode decreases monotonically with ω_h. This means that for wider waveguide widths the cutoff thickness is significantly reduced. At the same time, the values of the indistinguishability and the coupling efficiency achieved in our exploration with d = 800 nm and ω_h between 130 and 170 nm are maintained for higher ω_h. As an example, for our fabricated structure we chose ω_h = 360 nm, that demands a thickness of d =130 nm for single mode operation. This specific structure was fabricated with the aim of depositing a $WeSe_2$ monolayer on top, requiring a wider platform support for optimal flake adherence. With ω_s = 38 nm, and $\gamma^* = 100$ the expected indistinguishability with this emitter and cavity parameters is I = 0.95 according to our model.

The device is fabricated with CMOS compatible process. We first deposit a layer of 1045nm silicon dioxide with plasma enhanced chemical vapor deposition (PECVD) on a silicon substrate. Then we deposit 130nm silicon nitride with low pressure chemical vapor deposition (LPCVD). We pattern the device with e-beam lithography and HSQ resist. The pattern is etched with inductively coupled plasma reactive ion etching (ICP-RIE) and $CHF_3/N_2/O_2$ chemistry. At last, we dip the wafer in HF solution (1:100) to remove HSQ residue.

References

1. Grange, T., Hornecker, G., Hunger, D., Poizat, J. P., Gérard, J. M., Senellart, P., & Auffèves, A. (2015). Cavity-funneled generation of indistinguishable single photons from strongly dissipative quantum emitters. Physical review letters, 114(19), 193601.
2. Lumerical Inc. https://www.lumerical.com/products/ .
3. Novotny, L., & Hecht, B. (2012). Principles of nano-optics. Cambridge university press.
4. Liu, Y., Kong, M., & Jiang, Y. (2015). Transverse magnetic modes in planar slot waveguides. JOSA B, 32(10), 2052-2060.
5. Epping, J. P., Hellwig, T., Hoekman, M., Mateman, R., Leinse, A., Heideman, R. G., ... & Boller, K. J. (2015). On-chip visible-to-infrared supercontinuum generation with more than 495 THz spectral bandwidth. *Optics express*, *23*(15), 19596-19604.
6. Pfeiffer, M. H., Kordts, A., Brasch, V., Zervas, M., Geiselmann, M., Jost, J. D., & Kippenberg, T. J. (2016). Photonic Damascene process for integrated high-Q microresonator based nonlinear photonics. *Optica*, *3*(1), 20-25.
7. Luke, K., Poitras, C. B., & Lipson, M. (2013, July). Overcoming SiN film stress limitations for high quality factor ring resonators. In *2013 IEEE Photonics Society Summer Topical Meeting Series* (pp. 64-65). IEEE.
